# Detection of a novel *SETBP1* variant in a Chinese neonate with Schinzel–Giedion syndrome

**DOI:** 10.3389/fped.2022.920741

**Published:** 2022-09-06

**Authors:** Hansong Yang, Zhiyong Liu, Dongmei Chen, Weiru Lin, Lin Wang, Tianfeng Chen, Ruiquan Wang, Xialin Yan

**Affiliations:** ^1^Department of Neonatology, Quanzhou Maternity and Children's Hospital, Quanzhou, China; ^2^Department of Ultrasound, Quanzhou Maternity and Children's Hospital, Quanzhou, China; ^3^Xiamen Genokon Medical Technology Co., Ltd., Xiamen, China; ^4^Department of Radiology, Quanzhou Maternity and Children's Hospital, Quanzhou, China

**Keywords:** Schinzel-Giedion syndrome, *SETBP1*, neonate, phenotypes, neurodevelopmental delay

## Abstract

Schinzel–Giedion syndrome (SGS) is a multiple malformation syndrome characterized by typical facial features, severe neurodevelopmental delay, and multiple congenital abnormalities. SGS is associated with *de novo* pathogenic variants in the *SETBP1* gene. In specific, *SETBP1* variants in over 50 patients with classical or non-classical SGS were clustered within exon 4. A male Chinese neonate with dysmorphic facial features, nervous system disorders, and organ malformations at birth was examined in this study and long-term followed-up. Whole-exome sequencing was performed to identify any underlying pathogenic variants in the proband. Additionally, we reviewed the literature that documents the main clinical features and underlying variants of all patients genetically diagnosed with SGS. The neonate had a characteristic midface retraction, abnormal electroencephalogram waveforms, and genital abnormalities. The patient did not initially develop hydronephrosis or undergo a comprehensive skeletal assessment. Six months after birth, the patient had an epileptic seizure and experienced persistent neurodevelopmental delay with auditory and visual abnormalities. Color Doppler ultrasonography at 18 months revealed hydronephrosis and bilateral widening of the lateral ventricles. The patient died suddenly 20.5 months after birth. Whole-exome sequencing revealed a heterozygous *de novo* variant (c.2605A > G:p.S869G) in exon 4 degradation sequence in *SETBP1*. The reported *de novo* heterozygous variant in *SETBP1* (c.2605A > G:p.S869G) broadens the knowledge of the scientific community's on the possible SGS genetic alterations. To the best of our knowledge, this is the first report of *SETBP1* variant (c.2605A > G:p.S869G) in SGS. The clinical manifestations of neonatal SGS are atypical, and genetic testing is crucial for diagnosis. Long-term follow-up should be conducted after diagnosis to optimize the therapeutic interventions.

## Introduction

Schinzel–Giedion syndrome (SGS, OMIM 269150), also known as midface retraction syndrome, is an extremely rare autosomal dominant genetic disorder. It is characterized by neurodevelopmental delay, midface retraction, epilepsy, multiple congenital malformations, and an increased risk of cancer in children (1, 2). The specific prevalence of SGS remains unclear, but its contribution to reduced life expectancy in children is significant (3, 4).

In 2010, Hoischen et al. (5) reported that heterozygous *SETBP1* variants are associated with the development of SGS. *SETBP1*, located on chromosome region 18q21.1, encodes an oncogene-binding protein. SETBP1 protein has various biological functions, including binding to SET domains involved in the methylation of the lysine residues on histone tails. Its universal expression explains the multisystem SGS manifestations (6). The pathogenic missense variants in *SETBP1* associated with the classical SGS phenotype are confined to a hotspot region of 12 base pairs (bp) in exon 4. This gene sequence encodes 4 amino acids in the SKI homologous region of the SETBP1 protein (D868, S869, G870, and I871), which constitute the degron degradation sequence (3, 7). The SKI region containing SGS hotspots is a key region for substrate recognition by homologous SCF-β-TrCP E3 ubiquitin ligase; the deletion of this region can result in protein overexpression (8). Recent studies suggest that accumulation of SETBP1 protein in cells is intolerable. Antonyan et al. (9) propose assumption of the mechanisms of SETBP1 action by several different molecular complexes. Gene ontology analysis of dysregulated SETBP1 target genes indicates that they are also key controllers of visceral organ development and brain morphogenesis (10). However, how does the variant of *SETBP1* gene led to a variety of malformations in SGS patient is still unknown (9).

In 2008, Lehman et al. (2) reviewed 46 SGS cases. They proposed clinical diagnostic criteria based on developmental delay (excluding neonates) and typical facial features associated with hydronephrosis or characteristic skeletal malformations. The skeletal malformations are usually two or more, including a sclerotic skull base, wide occipital synchondrosis, increased cortical density or thickness, and broad ribs (2). Since the identification of *SETBP1* as a pathogenic gene in 2010, more than 50 molecularly diagnosed SGS cases have been reported (3, 4, 7, 11–16), including some non-classical cases. In 2018, Liu et al. (13) proposed revised diagnostic criteria for SGS and widened the phenotypic spectrum to include patients presenting with fewer phenotypic manifestations. However, they did not explain the correlation between the mutated genes and the phenotypes. The emergence of molecular diagnosis may result in more frequent reports of non-classical SGS. Moreover, variants in the *SETBP1* classic degradation sequence may produce non-classical SGS, such as in the patients reported by Sullivan et al. (4) and the Chinese patients reported by Lu et al. (15).

SGS is characterized by progressive changes, and its diagnosis in the neonatal period is difficult because of the relatively few phenotypes presented during this period. In fact, classical SGS has not yet been reported in China. In this study, we investigated the genetic characteristics of a male Chinese neonate having a *de novo SETBP1* variant. To the best of our knowledge, this is the first report of *SETBP1* variant (c.2605A > G:p.S869G) in SGS. Our study highlights the importance of molecular studies, early diagnosis, and long-term follow up for patients in order to optimize their treatment strategies.

## Materials and methods

### Study subject

A male neonate who was admitted to the neonatal intensive care unit of Quanzhou Maternity and Children's Hospital was included in this study. He exhibited dysmorphic facial features, weak sucking reflex, low muscle tone, and neurological disorders. We conducted long-term follow-up for his clinical condition. This study was approved by the Ethics Committee of Quanzhou Maternity and Children's Hospital. The patient's parents provided written informed consent to the study.

### Genetic analysis

#### Whole-exome sequencing and prediction of the mutated gene functions

Two milliliters of the peripheral blood collected from the patient were used (anticoagulant: EDTA), and whole-exome sequencing was performed using the blood sample by the Genokon Medical Laboratory (Xiamen, China). The Blood Genome Extraction Kit (Tiangen Biochemical Technology Co., Ltd., Beijing, China) was used to extract the total genomic DNA from peripheral blood leukocytes. The NGS Fast DNA Library Prep Set for Illumina (Beijing Kangwei Century Biotechnology Co., Ltd., Beijing, China) was used to construct a DNA library. The IDT xGen Lockdown Reagents kit (IDT, USA) was used for hybridization-based capture of the target and flanking regions using probes. After target enrichment, the NovaSeq 6000 high-throughput sequencer (Illumina, USA) was used to perform paired-end 150 bp sequencing, with a mean sequencing depth of 100X and sequencing coverage of 99 %. The raw data from high-throughput sequencing were subjected to software quality control to remove low-quality sequencing data. The short reads generated from sequencing were compared to the reference sequence of the human genome (GRCh37/hg19), and GATK software (https://software.broadinstitute.org/gatk/) was used to analyze the information on the mutated sites. The single nucleotide polymorphisms and insertion and deletion variants detected were annotated using ANNOVAR software (17). The common variants found in the 1000 Genomes Project and the ExAC and gnomAD databases were filtered out, including intergenic, upstream, downstream, intronic, and synonymous variants, and variants with a minor allele frequency > 1 %. Computer software, including REVEL (18), ClinPred (19), SIFT (20), Polyphen2 (21), PROVEAN (22), and MutationTaster, were used to predict the deleterious effects of each variant on the protein function (23). Exomiser (24) and Phenolyzer (25) software were used to perform genotype–phenotype analysis. Homology modeling was performed using the PyMOL software (www.pymol.org) to analyze changes in the three-dimensional structure, and evolutionarily conserved regions were analyzed using MEGA software (www.megasoftware.net). Finally, the pathogenicity assessment and genetic interpretation of candidate gene variants were performed according to the American College of Medical Genetics and Genomics (26) guidelines and criteria for variant classification.

#### Sanger sequencing

The upstream and downstream primers were designed according to the information on the candidate variant sites detected in the probands. Polymerase chain reaction was subsequently performed. Primer design.

*SETBP1*-F, 5′-TTTCAGTCACTTGTGGCGTCTT-3′;
*SETBP1*-R, 5′-TTCCGTTTCCTCTTGTGCTTTG-3′.

The purified polymerase chain reaction products were directly sequenced using the ABI 2720 DNA analyzer. The NCBI BLAST algorithm was used for sequence alignment. Additionally, variants and the main clinical features of all patients genetically diagnosed with SGS were reviewed. Data on these patients were retrieved from PubMed (http://www.ncbi.nlm.nih.gov/pubmed) using the search keywords “Schinzel–Giedion syndrome” or “SGS” and “*SETBP1*.”

## Results

### Clinical findings

**Figure 1:**
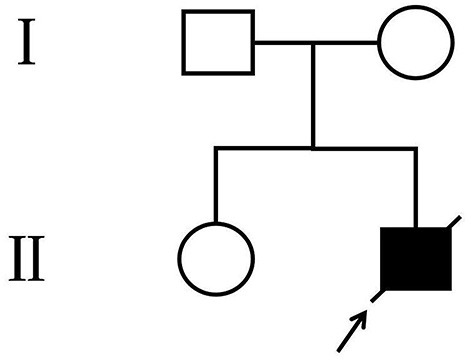
Pedigree of the family. The arrow denotes the proband and the hollow symbols represent the unaffected members.

A 3-day-old male infant was from a family in Quanzhou, Fujian province, China (Figure 1). The patient was admitted to the hospital owing to poor appetite and drowsiness since birth. The naturally conceived singleton infant was delivered by a G_2_P_2_ female *via* cesarean section owing to uterine scarring at 38^+6^ weeks of gestation. Color Doppler ultrasonography at 12 weeks of gestation indicated that the nuchal translucency (NT) of the fetus was 2.4 mm; non-invasive DNA testing showed no abnormalities. Ultrasonography at 23 weeks of gestation revealed bilateral choroid plexus cysts and strong punctate echoes in the left cardiac ventricle. Amniocentesis was conducted for chromosomal microarray analysis, and no abnormalities were found. There was no prenatal history of premature membrane rupture, fever, or fetal distress and no amniotic fluid, umbilical cord, or placental abnormalities. His Apgar score was 9-10-10, his birth weight was 3,300 g (47^th^ percentile), his height was 50 cm (48^th^ percentile), and he had a head circumference of 34 cm (38^th^ percentile). Three days after birth, the patient experienced poor appetite and was artificially fed with 10 mL formula milk once every 2–3 h. In addition, the infant exhibited drowsiness and a weak cry. However, he was responsive to plantar stimulation, and there was no fever, shortness of breath, and convulsion. Before admission, he did not receive treatment outside the hospital setting and was transported through the neonatal transport system to the neonatal intensive care unit.

**Figure 2:**
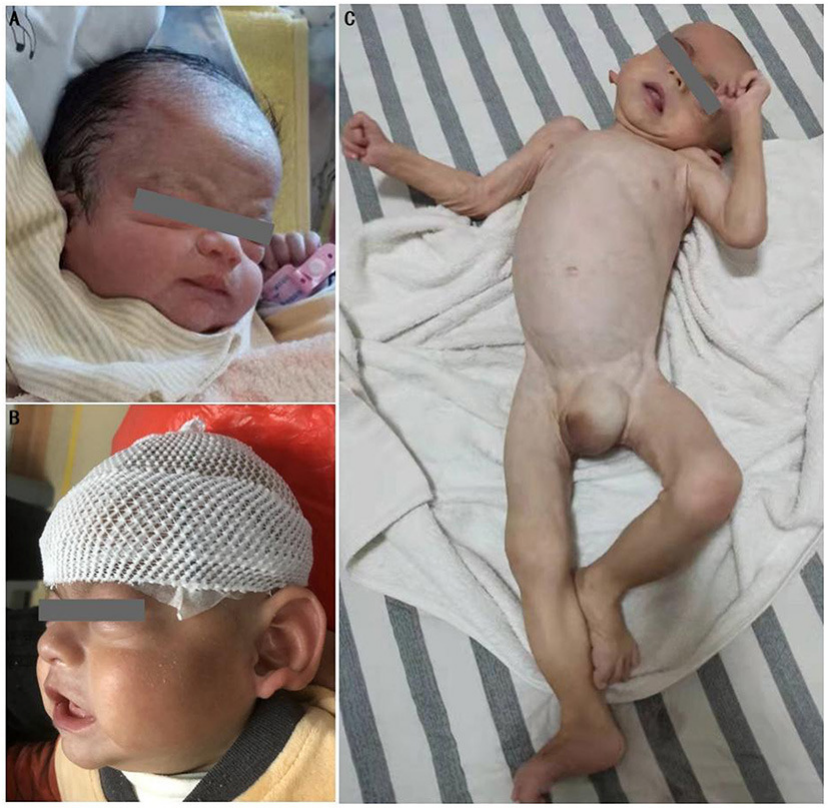
Physical deformities of the patient. **(A)** characteristic midface retraction, frontal bossing, “wide-spaced” eyes, low nasal bridge, and low-set ears at 1 day after birth; **(B)** notable abnormalities in auricles at 6 months after birth; **(C)** joint contractures of extremities, trunk incurvation, left oblique inguinal hernia, and genital abnormalities at 18 months after birth.

His physical examination upon admission showed stable vital signs, sanity, and stable breathing. However, he appeared drowsy, and although the infant responded by crying after 3-4 plantar stimulations, his cry was noticed to be weak. In addition, he had slightly low muscle tone, incomplete embrace reflex, weak sucking reflex, and simian crease across the palm of the right hand. The cardiopulmonary examination showed no abnormalities. Moreover, the anterior fontanelle was flat and soft. Unique facial features (Figures 2A,B) included mild midface retraction, frontal bossing, hypertelorism, low nasal bridge, low-set ears, and abnormal auricles. The testes were not palpated in the scrotum, and retraction of the foreskin revealed a micropenis. The patient's parents (non-consanguineous marriage) and elder sister were in good health, and there was no family history of genetic disorders, including structural malformations at birth and neurodevelopmental abnormalities.

**Figure 3:**
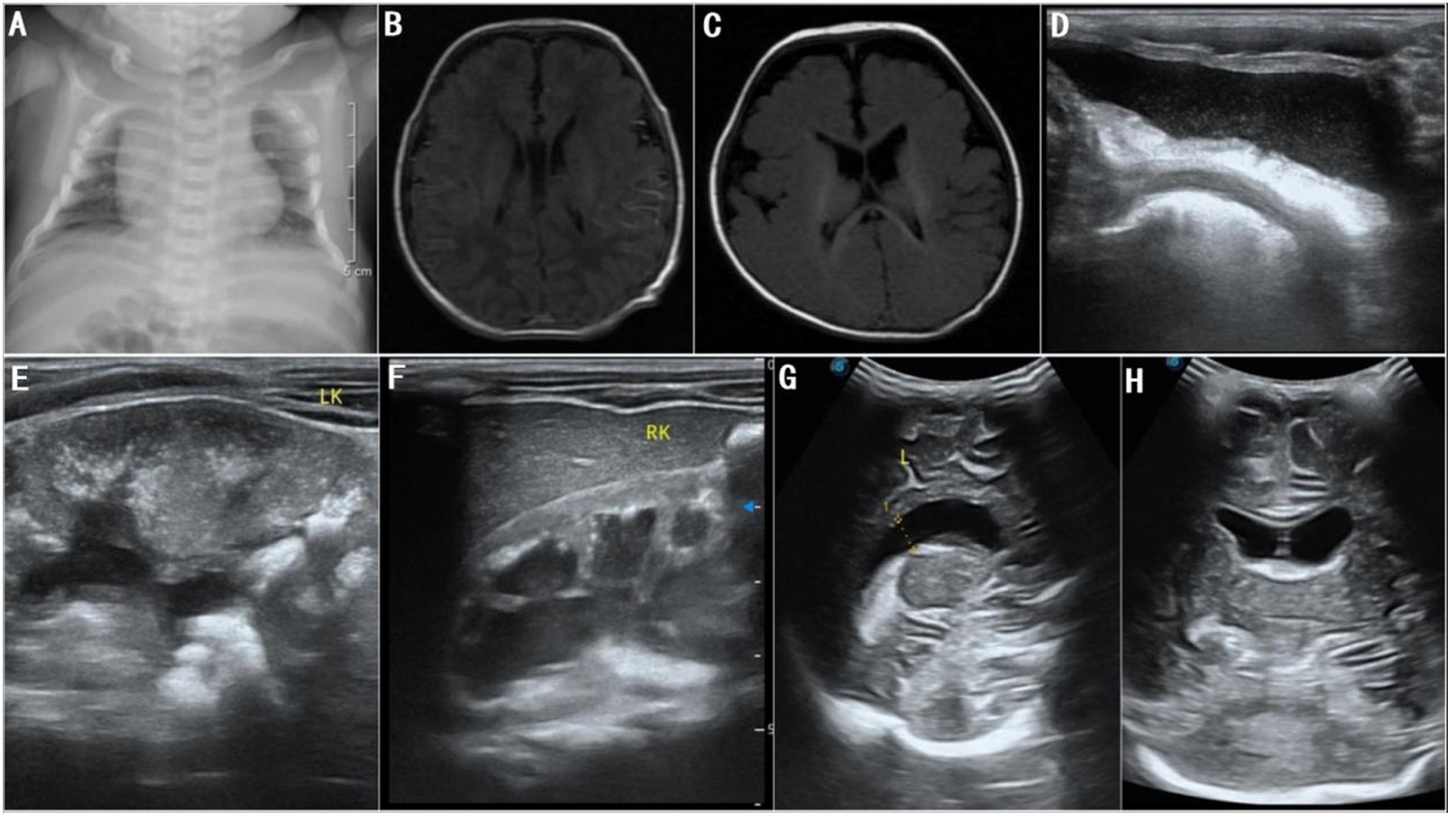
**(A)** Chest radiographs at 3 days after birth show broad bones from the 2^nd^ to the 5^th^ pair of anterior ribs and thoracic deformity; **(B)** brain MRI at 5 days after birth shows normal lateral ventricles; **(C)** brain MRI at 6 months after birth shows normal lateral ventricles; **(D–F)** color Doppler ultrasonography at 18 months after birth shows bilateral hydronephrosis, and deposition of calcium in bilateral renal parenchyma, bilateral renal pelvises, and bladder; **(G,H)** color Doppler ultrasonography at 18 months after birth shows widened bodies and anterior horns of lateral ventricles.

The patient received parenteral nutrition and performed sucking exercises for 7 days after admission and was discharged from the hospital after slow artificial total enteral feeding could be administered. During treatment, the patient had normal lab results for full biochemistry profile, routine blood, urine, and stool examinations, and thyroid function tests. Moreover, he had negative results for toxoplasmosis, rubella, cytomegalovirus, herpes simplex, and HIV (TORCH) antibody tests. In addition, tandem mass spectrometry screening of blood and urine samples and karyotype chromosome analysis showed normal results. Chest radiographs revealed broad bones between the 2^nd^ to the 5^th^ pair of anterior ribs and a thoracic deformity (Figure 3A). Color Doppler echocardiography showed no abnormality; however, color Doppler ultrasonography of the urinary system suggested bilateral cryptorchidism. Moreover, both ears did not respond to the rapid auditory brainstem response test. No ocular fundus abnormalities were observed; however, bilateral poor light reflexes in the eyes were observed on eye screening. His neonatal behavioral neurological assessment score was 36. Video electroencephalogram (EEG) showed mild discontinuity of the background EEG, distinct desynchronization in the left and right hemispheres, intermittent discharge of a few sharp waves, spike waves, and arrhythmic waves in the bilateral central and temporal regions during wakefulness and sleep. Plain brain magnetic resonance imaging (MRI) scan showed no abnormal manifestations (Figure 3B). Given the patient's unique facial features, genital abnormalities, and nervous system disorders, the possibility of a genetic disease was high in the differential diagnoses. The patient was eventually diagnosed with SGS 25 days after birth based on the results of whole-exome sequencing and clinical manifestations and was subsequently followed up.

After being discharged from the hospital, the patient underwent an ineffective home-based rehabilitation exercise program. Six months after birth, he experienced occasional bilateral shaking in lower limbs and occasional repeated bilateral blinking of the eyes; plain brain MRI scans did not show any structural abnormalities (Figure 3C). EEG revealed nearly continuous hypsarrhythmia in the sleep state and persistent lack of periodic changes in the background EEG activity in the active sleep/quiet sleep state; thus, a possible diagnosis of infantile spasms was considered. Considering the poor prognosis, the patient's parents opted for palliative treatment instead of antiepileptic therapies. Approximately 8 months after birth, shaking in lower limbs and eye blinking were not prominent. Nine months after birth, the patient gradually developed a left oblique inguinal hernia (Figure 2C), abdominal bloating, and an intestinal obstruction. Glycerin enemas were intermittently administered to relieve the constipation. At 18 months, the patient had extremely poor physical development with a bodyweight of 4,500 g, height of 65 cm, and head circumference of 41.2 cm (<1^st^ percentile for all three indicators). He also had a weak sucking reflex, inability to receive complementary foods, eruption of one tooth, irritability, high-pitched cry, joint contractures of the extremities, trunk incurvation (Figure 2C), inability to lift the head, insufficient lower limb support, inability to roll over, inability to track moving objects with both eyes, and no auditory response in both ears. The patient scored 8 points on the developmental quotient in the Gesell development scale and was classified as having an extremely severe mental deficiency. Color Doppler ultrasonography detected bilateral hydronephrosis, deposition of calcium in bilateral renal parenchyma, renal pelvises, and bladder (Figures 3D–F), widened bodies and anterior horns of lateral ventricles (Figures 3G,H), and absence of tumors in the abdominal cavity. Finally, the patient died suddenly 20.5 months after birth.

### Genetic findings

Whole-exome sequencing suggested a heterozygous variant (c.2605A > G:p.S869G) in exon 4 of *SETBP1* (NM_015559). This variant was a missense variant, i.e., the substitution of guanine (G) for adenine (A) on the 2605^th^ base pair, resulting in the substitution of the 869^th^ amino acid glycine for serine. The detected variant, with a Revel score of 0.641, was not found in many databases including the 1,000 Genomes Project, ExAC gnomAD and ClinVar databases. Other *silico* prediction, including ClinPred (score: 0.986), SIFT (score: 0.912), Polyphen2 (score: 0.998), PROVEAN (score: 0.717), and MutationTaster (score: 1), suggested the deleterious effects of this variant on protein function. There has been no previous report of such variants.

**Figure 4:**
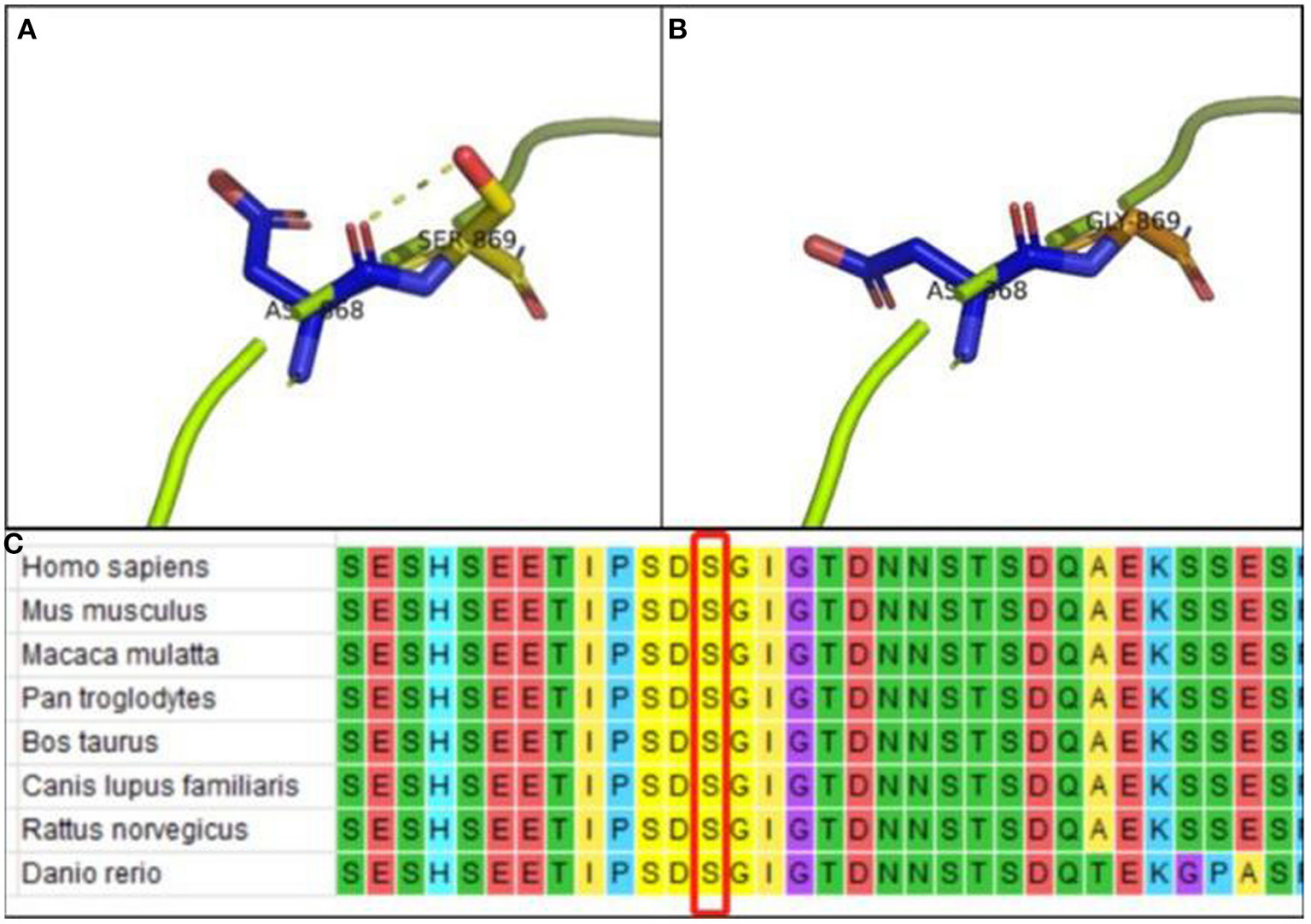
**(A)** PyMOL software analysis: Ser869 interacts with Asp868 *via* hydrogen bonding in wild-type p.Ser869 in SETBP1; **(B)** PyMOL software analysis: no hydrogen-bond interaction between Gly869 and Asp868 in mutant-type p.Ser869 in SETBP1, indicating that variants affect protein structure and function; **(C)** Alignment of the *SETBP1* sequences revealed that the amino acid residues at position 869 were strictly conserved.

Protein modeling showed that the interaction between Ser869 and Asp868 *via* hydrogen bonding maintained the stability of the protein structure of wild-type SETBP1 (Figure 4A). However, the *de novo* variant which resulted in substituting Gly869 for Ser869 resulted in the loss of the hydrogen-bonding interaction with Asp868 (Figure 4B). Hence, variants affecting the protein structure consequently affect the protein function. Alignment of the *SETBP1* sequences revealed that the amino acid residues at position 869 were strictly conserved (Figure 4C).

**Figure 5:**
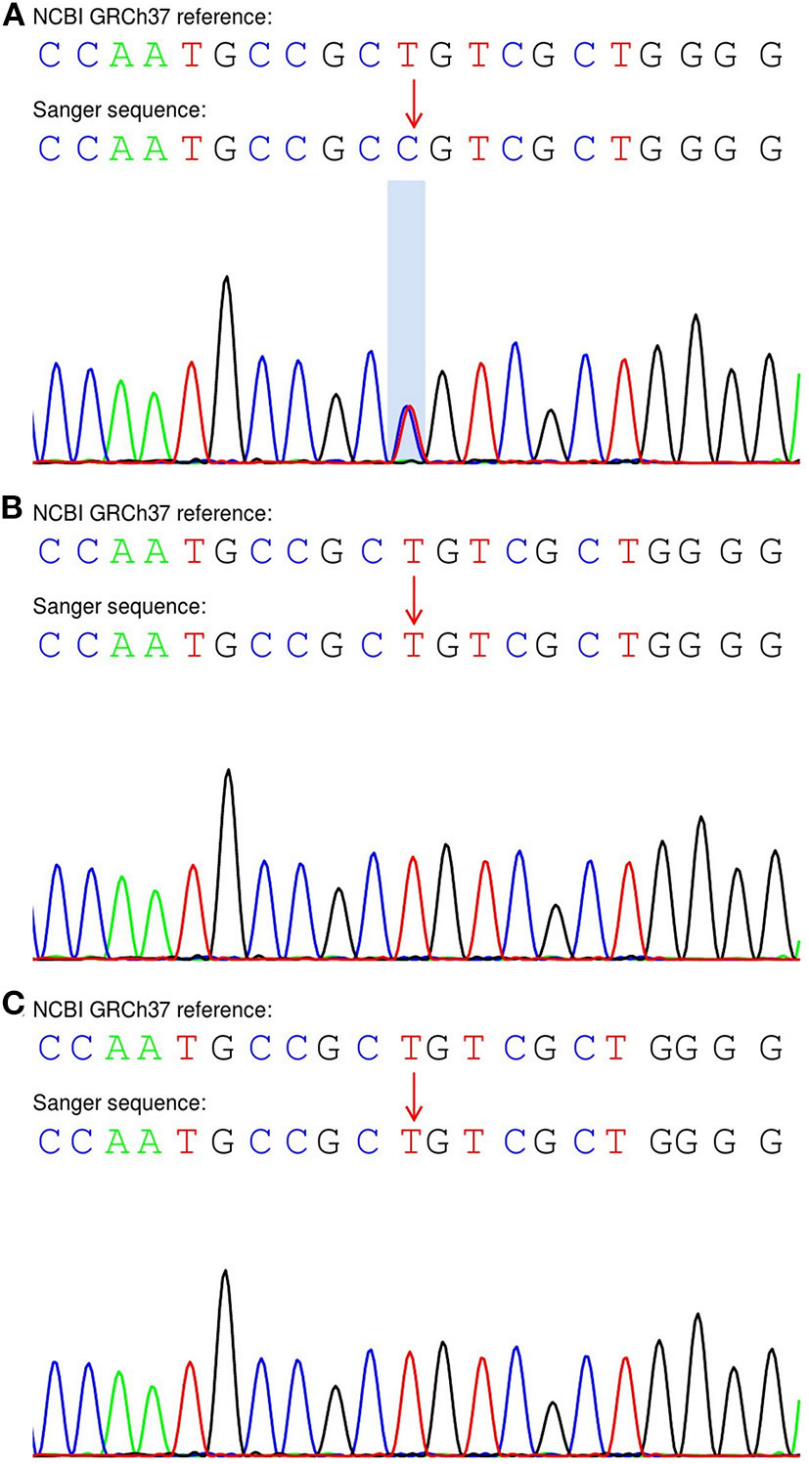
Sanger sequencing of the *SETBP1* gene in the proband and his parents. **(A)** The patient exhibited a heterozygous variant (c.2605A > G:p.S869G). **(B,C)** His unaffected parents carried the wild-type allele.

Sanger sequencing of the genome of the patient and his parents suggested that the patient had a *de novo* variant (Figure 5A); as both parents had the wild-type gene (Figures 5B,C). This variant was classified as pathogenic according to American College of Medical Genetics and Genomics guidelines (the supporting evidence for pathogenicity was PS2+PM1+PM2_Supporting+PM5_Strong+PP3).

The aforementioned data, the patient's clinical manifestations, and the *SETBP1* variant status (indicative of autosomal dominant genetic disorder) indicated that SGS was caused by a heterozygous variant (c.2605A > G:p.S869G) in *SETBP1*.

In addition to the reported newly diagnosed patient with SGS, we included in the cohort 59 patients who were previously diagnosed with SGS. 12 types of missense variants in exon 4 degron of *SETBP1* were observed in 54 of the 60 patients. The three most frequently detected variants were c.2602G>A (15/54), c.2608G>A (13/54), and c.2612T>C (14/54). The variants and the main clinical features of the 60 patients are presented in Table 1.

**Table 1:** *SETBP1* variants and the main clinical features of 60 patients clinically diagnosed with SGS.

**cDNA**	**Protein**	**Case**	**Midface** **retraction**	**Developmental** **delay**	**Hydronephrosis**	**Typical skeletal** **malformations**	**Seizures**	**Patients** **reported**
**Degron variants (868-871)**								
c.2602G > A	p.Asp868Asn	15	15/15	14/14	14/15	7/9	15/15	(3, 5, 12, 15, 27)
c.2602G > T	p.Asp868Thr	1	1/1	1/1	1/1	1/1	1/1	(3)
c.2603A > C	p.Asp868Ala	1	1/1	1/1	1/1	1/1	1/1	(5)
c.2605A > G	p.Ser869Gly	1	1/1	1/1	1/1	NA	1/1	This report
c.2605A > T	p.Ser869Cys	1	1/1	1/1	1/1	NA	1/1	(16)
c.2606G > A	p.Ser869Asn	1	1/1	1/1	1/1	1/1	1/1	(3)
c.2607C > G	p.Ser869Arg	1	1/1	1/1	1/1	NA	1/1	(3)
c.2608G > A	p.Gly870Ser	13	12/12	11/11	12/13	4/4	11/12	(3, 5, 7, 28–30)
c.2608G > T	p.Gly870Cys	1	1/1	1/1	1/1	0/1	1/1	(11)
c.2609G > A	p.Gly870Asp	3	3/3	3/3	3/3	2/2	3/3	(3, 5)
c.2612T > C	p.Ile871Thr	14	13/13	9/10	13/14	6/7	12/13	(3, 5, 31, 32)
c.2612T > G	p.Ile871Ser	2	2/2	2/2	1/2	NA	1/1	(4, 33)
Total		54	52/52	46/47	50/54	22/26	50/52	
**Non-degron variants**								
c.1181_1184insA	p.Glu394GlufsX	1	1/1	1/1	0/1	0/1	1/1	(13)
c.2572G > A	p.Glu858Lys	1	1/1	1/1	0/1	NA	1/1	(14)
c.2584G > A	p.Glu862Lys	1	1/1	1/1	0/1	NA	0/1	(3)
c.2601C > A	p.Ser867Arg	2	2/2	1/1	0/1	NA	2/2	(3, 27)
c.2618C > T	p.Thr873Ile	1	1/1	1/1	0/1	NA	0/1	(3)
Total		6	6/6	5/5	0/5	0/1	4/6	

## Discussion

The genotype–phenotype correlations for *SETBP1* variants are clinically relevant yet extremely complicated (3, 14). Studies have shown that *SETBP1* variants causing SGS had a gain-of-function or a dominant-negative effect, whereas haploinsufficiency or loss-of-function *SETBP1* variants caused a milder phenotype (27, 34). Recurrent missense variants at codons 868–871, forming the critical consensus sequence of the degradation signal, have been associated with classical SGS. Patients with missense variants near the degron sequence (codons 862, 867, and 873) exhibit a milder SGS phenotype, and the clinical overlap with the classical SGS phenotype is related to the position of variant (3, 4, 14). To the best of our knowledge, this is the first report of *SETBP1* variant (c.2605A > G:p.S869G) in SGS. Being highly conserved in different species, S869 might have an important biological role (7). Furthermore, protein model analysis of the variant suggests that it might affect the protein structure, thus causing diseases. We collected the data of 60 patients with molecularly diagnosed SGS, and 12 types of missense variants were observed in the 54 patients having degradation sequence variants. The most common variants were c.2602G > A, c.2608G > A, and c.2612T > C, suggesting that they might be “hotspot variants” for classical SGS. Variants of S869 are relatively rare in exon 4 degron (3, 16); therefore, reports of novel and rare variants may facilitate analysis of the genotype–phenotype correlations and the study of their underlying mechanisms.

The common non-specific SGS symptoms like abnormal facial development, genital abnormalities, reduced sucking ability, decreased muscle tone, and EEG waveform abnormalities render the diagnosis of SGS in neonatal wards challenging (7). Hence, molecular diagnosis is crucial when several genetic syndromes can be diagnosed. The neonate in this report did not initially present with hydronephrosis, and his cranial bones were not surgically evaluated because the evaluation was not particularly helpful for treatment. Based on the diagnostic criteria proposed by Lehman et al. (2) and the presence of a heterozygous variant in *SETBP1* (c.2605A > G:p.S869G) confirmed by molecular analysis, we deemed that the patient may have “non-classical” SGS. During the 18-month follow-up, color Doppler ultrasonography revealed bilateral hydronephrosis; therefore, the diagnosis was changed to classical SGS. Phenotypic changes are common, particularly in infants who have undergone an early molecular diagnosis.

Previous studies have suggested that hydronephrosis may not necessarily be a mandatory diagnostic criterion in SGS (3). Among the 54 patients with SGS having variants in the degron sequence, four patients did not exhibit hydronephrosis. Based on our report, hydronephrosis should have shown a slower development. Progressive hydronephrosis occurred in long-term survivors, but the patients did not develop renal failure (33). The four types of skeletal changes included in the diagnostic criteria (2) did not significantly impact the patients' quality of life. In addition, <50 % of patients with either classical or non-classical SGS underwent comprehensive skeletal assessments. Therefore, evaluating *SETBP1* variants is required in infants with midface retraction and other body system abnormalities. In the diagnostic criteria updated by Liu et al. (13), the diagnosis of type III (simple) SGS was deemed inappropriate, as patients with *SETBP1* variant and developmental delay are more likely to have autosomal dominant intellectual disability type 29 (OMIM ^*^ 616078) (34, 35). Type II SGS is more likely to be categorized as non-classical SGS if the *SETBP1* variant is located in or adjacent to the degron sequence.

The incidence of neurodevelopmental delay (51/52) and epilepsy (54/58) was extremely high among the patients. Accordingly, SGS might be considered a type of developmental and epileptic encephalopathy. A previous report showed that two patients with SGS exhibited progressive brain atrophy (33). In our report, the patient's initial brain MRI scans did not reveal structural brain abnormalities. During a recent follow-up visit, the patient's family members did not approve carrying out cranial MRI and CT scans, but color Doppler ultrasonography showed preliminary enlargement of cerebral ventricles. This patient presented with mild EEG waveform abnormalities during the neonatal period and had an epileptic seizure 6 months after birth. Hence, a possible diagnosis of infantile spasms based on EEG was considered. The patient's family members opted for palliative treatment. Although the epileptic seizures stopped 8 months after birth, the patient suffered from extremely severe neurodevelopmental delay, which might be related to not receiving active treatment. In a previous study, a patient had a variant in the same base pair (2605^th^) complicated by refractory epilepsy, which was controlled by the administration of topiramate and cannabidiol (16). According to existing reports, the longest duration of survival in a patient with SGS was 15 years (27); therefore, active treatment and home-based care are essential. Sullivan et al. (4) reported a patient with SGS having a weak performance despite having a missense variant in the degron region. Moreover, a patient reported by Lu et al. (15) experienced an improvement in language development after treatment. Therefore, the medical team should be cautious about their prognostic and genetic interpretations in younger SGS patients.

Our study has some limitations. We could not perform a comprehensive assessment that included complete skeletal assessments, blood tests for tumor markers, and assessment of the patient's response to pharmacotherapies and rehabilitation exercises owing to the rejection of the patient's parents to carry out these assessments. This study summarized the variants and the main clinical features of patients who are genetically diagnosed with SGS. Further analysis is required to evaluate the impact of different variants on the clinical phenotypes and prognoses.

In summary, we reported the clinical and genetic characteristics of the first Chinese neonate with classical SGS, possibly caused by a *de novo* heterozygous *SETBP1* variant (c.2605A > G:p.S869G). The neonate did not exhibit the typical phenotypes of SGS in the early neonatal period, making the diagnosis challenging. Long-term follow-up should be conducted after molecular diagnosis of SGS to optimize the therapeutic strategies.

## Data availability statement

The datasets for this article are not publicly available due to concerns regarding participant/patient anonymity. Requests to access the datasets should be directed to the corresponding authors.

## Ethics statement

The studies involving human participants were reviewed and approved by Ethics Committee of Quanzhou Maternity and Children's Hospital. Written informed consent to participate in this study was provided by the participants' legal guardian/next of kin. Written informed consent was obtained from the individual(s), and minor(s)' legal guardian/next of kin, for the publication of any potentially identifiable images or data included in this article.

## Author contributions

HY and ZL conceived the study and wrote the first draft of the manuscript. XY and RW helped critically revise the manuscript for important intellectual content and were the mentors who designed and guided the research study. LW carried out the variant analyses. DC, WL, and TC oversaw patient care and collected the clinical data. All authors contributed to manuscript revision, read, and approved the final submitted version of the manuscript.

## Funding

This work was funded by the Cooperative Innovation Project of Quanzhou Maternity and Children's Hospital and Huaqiao University (Grant No. 2021YX001).

## Conflict of interest

LW was employed by Xiamen Genokon Medical Technology Co., Ltd. The remaining authors declare that the research was conducted in the absence of any commercial or financial relationships that could be construed as a potential conflict of interest.

## Publisher's note

All claims expressed in this article are solely those of the authors and do not necessarily represent those of their affiliated organizations, or those of the publisher, the editors and the reviewers. Any product that may be evaluated in this article, or claim that may be made by its manufacturer, is not guaranteed or endorsed by the publisher.
